# Workshop: health technology assessment of integrated home care for frial elder somatic patients

**Published:** 2012-09-04

**Authors:** Torben Larsen, Silvina Santana

**Affiliations:** CAST/SDU Denmark, Albert Alonso, Fundacio Privada Clinic per la Recerca Biomedica Barcelona, Spain; Universidade de Eveiro Portugal, Luca Greselin, Region Veneto, Italy

**Keywords:** health technology assessment, patient satisfaction, effectiveness, quality, access, economy

## Abstract

**Background:**

The fragmented delivery of healthcare and social services as advanced by WHO 2002.

**Objectives:**

This project of international collaboration assesses integrated home care (IHC) for frail elder somatic patients as compared to usual hospital care.

**Methods:**

The HTA follows the special application for Tele-medicine (MAST). An introductory literature review identified stroke, heart failure (HF) and COPD as prototypes of IHC. Pre-existing evidence has been complemented by additional trials and surveys.

**Results:**

1. Definition/organization of IHC:

1. Is carried out by a multidisciplinary team visiting the home
2. Considers effectiveness, quality, access and user satisfaction in an economic way and uses Tele-facilities as far as they serve these goals
3. Has finance across organizational settings.

2. Clinical effectiveness of IHC for moderately disabled patients by 6–12 months follow-up:

Stroke: In 14 randomized trials (n=2139) intervention patients were by meta-analysis significantly less likely (p=0.001) to be dead or dependent compared with conventional care.

HF: 2 RCT (n=386) demonstrate each a significant reduction of all-cause readmissions (p=0.003 and p=0.001).

COPD: 5 studies (2 RCT, 2 cohorts and 1 CT) (n=1249) demonstrate each a significant reduction in readmissions/total admission days (p<0.05).

3. Health economic evaluation:

For each selected condition the first year benefit surmounts the costs of intervention using the Dutch Standardization by Oostenbrink as a common price catalogue across resources/trials/countries.

4. Patient satisfaction:

Focus group interviews confirm literature findings of very good satisfaction by IHC both among patients/carers and health professionals.

**Discussion:**

Calculated net savings of 1450€ per patient in IHC are not supposed to materialize in ‘cool’- cash but enables local negotiation of adapted solutions with a minimum of national legislation/finance (Meso-strategy of dissemination).

**Implications:**

IHC is an approach to clinical continuity for a majority of frail elder somatic patients.

Powerpoint presentation available at http://www.integratedcare.org at congresses – San Marino – programme.

